# Role of colectomy in the management of appendiceal tumors: a retrospective cohort study

**DOI:** 10.1186/s12876-023-03019-4

**Published:** 2023-11-17

**Authors:** Victoria A. Marks, Daniel Kerekes, Samuel Butensky, Nita Ahuja, Caroline Johnson, Kiran Turaga, Sajid A. Khan

**Affiliations:** 1 Department of Surgical Oncology, Yale School of Medicine, Yale University School of Medicine, 330 Cedar Street, FMB130, New Haven, CT 06520 USA; 2 Yale School of Public Health, 60 College St, New Haven, CT 06510 USA

**Keywords:** Appendix, Appendiceal neoplasms, Appendectomy, Hemicolectomy, Survival analysis

## Abstract

**Background:**

Appendiceal tumors represent a range of histologies that vary in behavior. Recommendations for treatment with appendectomy versus right hemicolectomy (RHC) for different tumor types are evolving and sometimes conflicting. This study sought to characterize variation in the United States around surgical treatment of major appendiceal tumor types over time and describe differences in outcomes based on procedure.

**Methods:**

Patients diagnosed with appendiceal goblet cell adenocarcinoma (GCA), mucinous adenocarcinoma, neuroendocrine neoplasm (NEN), or non-mucinous adenocarcinoma from 2004–2017 were identified in the National Cancer Database. Trends in RHC over time and predictors of RHC were identified. Surgical outcomes for each histologic type and stage were compared.

**Results:**

Of 18,216 patients, 11% had GCAs, 34% mucinous adenocarcinoma, 31% NENs, and 24% non-mucinous adenocarcinoma. Rate of RHC for NEN decreased from 68% in 2004 to 40% in 2017 (*p* = 0.008) but remained constant around 60–75% for other tumor types. Higher stage was associated with increased odds of RHC for all tumor types. RHC was associated with higher rate of unplanned readmission (5% vs. 3%, *p* < 0.001) and longer postoperative hospital stay (median 5 days vs. 3 days, *p* < 0.001). On risk-adjusted analysis, RHC was significantly associated with increased survival versus appendectomy for stage 2 disease of all tumor types (HRs 0.43 to 0.63) and for stage 1 non-mucinous adenocarcinoma (HR = 0.56).

**Conclusions:**

Most patients with appendiceal tumors undergo RHC, which is associated with increased readmission, longer length of stay, and improved survival for stage 2 disease of all types. RHC should be offered selectively for appendiceal tumors.

## Introduction

Appendiceal cancer is a rare form of malignancy, with an incidence of approximately 1 per 100,000 persons in the United States annually [1]. Despite low case numbers, these tumors demonstrate significant heterogeneity, and the different histologic types vary widely in clinical presentation, response to therapy, and prognosis [2–4]. Classification of any appendiceal tumor is therefore essential to optimizing treatment and patient outcomes.

Appendiceal tumors are discovered either incidentally on cross-sectional imaging or after appendectomy performed for appendicitis. If discovered first on imaging, appendectomy is nearly universally utilized for tissue diagnosis. In rare cases, an appendiceal tumor may be discovered on colonoscopy and the diagnosis is made via endoscopic biopsy. However the diagnosis is made, the histopathology of the tumor directs definitive management. Appendiceal cancers can be epithelial or non-epithelial in origin. Epithelial tumors include low- and high-grade appendiceal mucinous neoplasms (noninvasive), mucinous adenocarcinoma, signet-ring cell adenocarcinoma (an aggressive, poorly-differentiated subtype of mucinous adenocarcinoma [5]), and non-mucinous (also called colonic-type or intestinal-type) adenocarcinoma. The most common non-epithelial appendiceal tumor is neuroendocrine neoplasm [6], which is further characterized as well- or moderately-differentiated (“carcinoid tumor” or “neuroendocrine tumor”) versus poorly-differentiated (“neuroendocrine carcinoma”). Some tumors demonstrate features of both epithelial and non-epithelial tissue: the most common of these in the appendix is goblet cell adenocarcinoma (previously called goblet cell carcinoid or adenocarcinoid due to neuroendocrine features, but since found to behave more like adenocarcinoma) [7, 8].

As evidenced by this wide-ranging terminology, the characterization and classification of this diverse disease have been refined over time. These modifications in classification have in turn been accompanied by evolutions in management recommendations, leading to confusion about the appropriate treatment for a patient presenting with an appendiceal tumor. While negative margin surgical resection has remained the cornerstone of management for nearly all appendiceal tumors, recommendations around the extent of resection –appendectomy versus right hemicolectomy (RHC) – have varied over time and by guideline.

For example, for goblet cell adenocarcinoma of the appendix, current American Society of Colon and Rectum Surgeons (ASCRS) guidelines and North American Neuroendocrine Tumor Society (NANETS) guidelines recommend formal oncologic resection with RHC for all patients [9, 10]. However, other studies and experts have advocated for simple appendectomy for low-risk cases of this disease, citing an apparent lack of disease-free survival advantage from RHC in real-world data [11–14]. Still others have suggested RHC does not go far enough and have recommended colectomy be accompanied by bilateral salpingo-oophrectomy for all female patients with this disease, in light of a described propensity to metastasize to the ovaries [15].

Management recommendations in the literature for almost all appendiceal tumor types are confusing at best and outright conflicting at worst [8, 9]. The dynamic, fragmented, and sometimes ambiguous state of the science and recommendations around appropriate management of these tumors introduces the opportunity for significant practice variation with respect to extent of surgical resection. This may lead to treatment inappropriate for an individual’s tumor biology and prognosis with undertreatment by appendectomy or overtreatment by hemicolectomy.

An understanding of how appendiceal tumors have been surgically managed over time in practice and of the outcomes related to differences in management is necessary to inform comprehensive and specific treatment recommendations. This study therefore sought to characterize variation in the United States around surgical treatment of major appendiceal tumor types over the past 15 years and to describe differences in outcomes based on this variation. We hypothesized that rates of RHC have decreased over time in light of studies identifying low-risk subgroups of these populations that do not benefit from RHC, and we suspected there may still be a misalignment in procedural aggressiveness and survival advantage for some patients.

## Methods

### Database and study population

In this retrospective cohort study, we utilized the National Cancer Database (NCDB), a national clinical oncology registry sponsored by the American College of Surgeons Commission on Cancer and the American Cancer Society [16]. This database represents more than 70% of newly diagnosed cancer cases in the United States and is therefore useful for studying practice patterns of rare tumors such as those of the appendix.

**Fig. 1:**
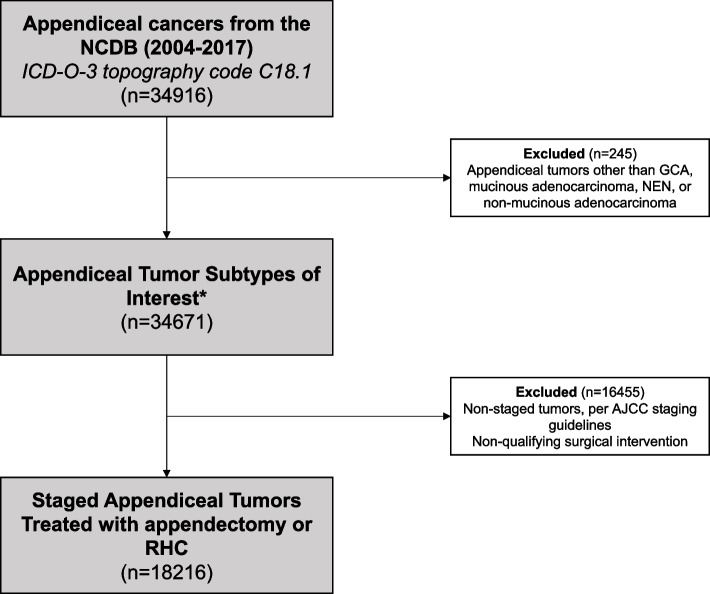
CONSORT diagram. * Histology codes 8013, 8010, 8020, 8140, 8144, 8210–1, 8220, 8240–6, 8248–9, 8255, 8260–3, 8310, 8323, 8420, 8430, 8440–1, 8460, 8470–2, 8480–1, 8490, 8560, 8574, 9015. AJCC: American Joint Committee on Cancer; GCA: Goblet Cell Adenocarcinoma; ICD-O-3: International Classification of Diseases for Oncology Third Addition; NCDB: National Cancer Database; NEN: Neuroendocrine Neoplasm; RHC: Right Hemicolectomy

We identified cases of appendiceal tumors between 2004 and 2017 from the 2017 “colon” Participant User File using the International Classification of Diseases for Oncology Third Addition (ICD-O-3) topographical code C18.1 [16, 17]. We selected histologies reflecting goblet cell adenocarcinoma (GCA), mucinous adenocarcinoma (including signet-ring adenocarcinoma), neuroendocrine neoplasm (NEN), and non-mucinous adenocarcinoma, consistent with prior studies (histology codes 8243; 8430, 8470–2, 8480–1, 8490, 9015; 8013, 8240–2, 8244–6, 8248–9; 8010, 8020, 8140, 8144, 8210–1, 8220, 8255, 8260–3, 8310, 8323, 8420, 8440–1, 8460, 8560, 8574, respectively) [1, 18–22]. Only patients treated with partial or total appendectomy or right hemicolectomy were included. Tumors not staged using American Joint Committee on Cancer (AJCC) staging guidelines were excluded (Fig. 1).

### Key study characteristics and outcomes

Demographic information collected included sex, age, race, Spanish/Hispanic origin, insurance status, median income quartile, local educational level, Charlson-Deyo Comorbidity Index, year of diagnosis, facility type, and facility location. Clinical information extracted included AJCC stage, tumor grade, lymphovascular invasion, and type of operation. Outcomes of interest were surgical margins, 30-day mortality, 90-day mortality, length of hospital stay following surgical resection, and readmission within 30 days of surgical discharge. Patients were stratified by histologic type for all analyses.

### Statistical analysis

Trends in surgical management over time for each histologic type were assessed. Baseline demographic and clinical characteristics stratified by histologic type were compared using chi-squared tests. A multivariable logistic regression adjusting for age (< 65 or ≥ 65), Charlson-Deyo score (0, 1, or ≥ 2), year of diagnosis (binary variable split at ≥ 2011), facility type, facility location, AJCC stage, grade, lymphovascular invasion, sex, ethnicity, race, insurance status, and urbanicity of facility was performed to determine predictors of RHC.

The outcomes of surgical margins, 30-day mortality, 90-day mortality, postoperative length of stay, and readmission within 30 days were compared for patients undergoing appendectomy versus RHC for each histologic type and for the study group overall. Two-tailed independent samples t-tests were used to compare length of stay and chi-squared tests were used to compare all other outcomes. Risk-adjusted long-term overall survival after appendectomy and after RHC for each stage of each histologic type were then compared using a multivariable Cox proportional hazards regression adjusting for the same variables used in the logistic regression. Survival information for patients diagnosed in 2017 was not available at the time of primary analysis and these patients were excluded from the Cox regression, as were any other patients with unknown survival. Missing values for predictor variables were assigned to a discrete category “unknown” to assess the association of missingness with the outcomes studied.

For all statistical tests, *p* < 0.05 was considered significant. Data were analyzed using IBM SPSS Statistics, version 28 (IBM Corporation, Armonk, New York).

As the NCDB is a deidentified database, study approval and informed consent were waived by the Institutional Review Board of Yale University.

## Results

### Practice patterns by type of operation

In total, there were 18,216 appendiceal tumors in the study group, including 1,970 (11%) GCA, 6,219 (34%) mucinous adenocarcinoma, 5,603 (31%) NEN, and 4,424 (24%) non-mucinous adenocarcinoma tumors. A majority of all patients were treated with RHC (60%). Overall, RHC was most frequently performed in GCA (69%) and least frequently performed in NEN (46%) (Table 1).

**Table 1:** Patient and tumor characteristics of the study cohort stratified by histologic subtype and operation

Characteristic	Goblet Cell n (%)		Mucinous n (%)		Neuroendocrine n (%)		Non-mucinous n (%)	
	**Appy**	**RHC**	**Appy**	**RHC**	**Appy**	**RHC**	**Appy**	**RHC**
**Sex**	*p* = 0.336		***p* < 0.001**		***p* = 0.007**		*p* = 0.991	
Male	303 (50)	658 (48)	844 (39)	1899 (47)	1165 (38)	1070 (42)	748 (53)	1578 (53)
Female	298 (50)	711 (52)	1302 (61)	2174 (53)	1880 (62)	1488 (58)	675 (47)	1423 (47)
**Age**	0.862		0.344		** < 0.001**		**0.002**	
< 65	431 (72)	987 (72)	1410 (66)	2627 (64)	2507 (82)	1828 (71)	727 (51)	1685 (56)
≥ 65	170 (28)	382 (28)	736 (34)	1446 (36)	538 (18)	730 (29)	696 (49)	1316 (44)
**Race**	0.561		0.404		**0.022**		0.426	
White	532 (89)	1219 (89)	1831 (85)	3508 (86)	2677 (88)	2226 (87)	1183 (83)	2508 (84)
Black	51 (8)	120 (9)	200 (9)	378 (9)	235 (8)	242 (9)	186 (13)	361 (12)
Other or Unknown	18 (3)	30 (2)	115 (5)	187 (5)	133 (4)	90 (4)	54 (4)	132 (4)
**Spanish/Hispanic Origin**	0.538		0.403		** < 0.001**		0.247	
Non-Spanish/Hispanic	573 (95)	1296 (95)	1917 (89)	3682 (90)	2741 (90)	2378 (93)	1307 (92)	2721 (91)
Spanish/Hispanic	9 (1)	31 (2)	146 (7)	247 (6)	240 (8)	114 (4)	72 (5)	157 (5)
Unknown	19 (3)	42 (3)	83 (4)	144 (4)	64 (2)	66 (3)	44 (3)	123 (4)
**Insurance Status**	0.574		0.393		** < 0.001**		0.204	
Private	351 (58)	814 (59)	1213 (57)	2271 (56)	1922 (63)	1470 (58)	641 (45)	1428 (48)
Medicare/Medicaid/Government	223 (37)	480 (35)	844 (39)	1600 (39)	902 (30)	968 (38)	723 (51)	1441 (48)
None	21 (4)	52 (4)	58 (3)	143 (4)	188 (6)	88 (3)	38 (3)	97 (3)
Unknown	6 (1)	23 (2)	31 (1)	59 (1)	33 (1)	32 (1)	21 (2)	35 (1)
**Urbanicity**	0.821		0.557		**0.013**		**0.007**	
Metropolitan	505 (84)	1136 (83)	1751 (82)	3334 (82)	2648 (87)	2150 (84)	1217 (86)	2466 (82)
Urban	73 (12)	183 (13)	223 (10)	440 (11)	292 (10)	288 (11)	159 (11)	370 (12)
Rural	8 (1)	14 (1)	25 (1)	54 (1)	34 (1)	43 (2)	15 (1)	49 (2)
Unknown	15 (3)	36 (3)	147 (7)	245 (6)	71 (2)	77 (3)	32 (2)	116 (4)
**Median Income (quartiles)**	0.341		0.374		0.068		0.694	
< $40,227	86 (14)	177 (13)	317 (15)	541 (13)	366 (12)	329 (13)	213 (15)	427 (14)
$40,227-$50,353	110 (18)	236 (17)	377 (18)	716 (18)	497 (16)	459 (18)	261 (18)	562 (19)
$50,354-$53,332	107 (18)	288 (21)	420 (20)	864 (21)	654 (22)	513 (20)	305 (21)	658 (22)
≥ $63,333	215 (36)	506 (37)	803 (37)	1507 (37)	1186 (39)	934 (37)	498 (35)	1011 (34)
Unknown	83 (14)	162 (12)	229 (11)	445 (11)	342 (11)	323 (13)	146 (10)	343 (11)
**No High School Degree (quar.)**	0.389		0.417		0.329		0.328	
≥ 17.6%	93 (16)	184 (13)	352 (16)	686 (17)	473 (16)	370 (14)	220 (16)	472 (16)
10.9%-17.5%	125 (21)	318 (23)	472 (22)	809 (20)	628 (21)	550 (22)	341 (24)	665 (22)
6.3%-10.8%	142 (24)	347 (25)	543 (25)	1061 (26)	823 (27)	665 (26)	360 (25)	805 (27)
< 6.3%	159 (27)	360 (26)	554 (26)	1076 (26)	784 (26)	653 (26)	361 (25)	721 (24)
Unknown	82 (14)	160 (12)	225 (11)	441 (11)	337 (11)	320 (13)	141 (10)	338 (11)
**Charlson-Deyo Score**	0.066		0.332		** < 0.001**		0.084	
0	485 (81)	1040 (76)	1751 (82)	3275 (80)	2632 (86)	2040 (80)	1075 (76)	2245 (75)
1	87 (14)	242 (18)	309 (14)	604 (15)	303 (10)	381 (15)	226 (16)	540 (18)
≥ 2	29 (5)	87 (6)	86 (4)	194 (5)	110 (4)	137 (5)	122 (9)	216 (7)
**Year of Diagnosis**	0.298		0.437		** < 0.001**		**0.038**	
2004–2010	90 (15)	181 (13)	651 (30)	1197 (29)	264 (9)	369 (14)	330 (30)	783 (26)
2011–2017	511 (85)	1188 (87)	1495 (70)	2876 (71)	2781 (91)	2189 (86)	783 (70)	2218 (74)
**Facility Type**	0.088		0.146		** < 0.001**		**0.048**	
Community	82 (14)	150 (11)	119 (6)	264 (7)	165 (5)	189 (7)	137 (10)	302 (10)
Comprehensive Community	238 (40)	512 (37)	728 (34)	1317 (32)	821 (27)	858 (33)	630 (44)	1225 (41)
Academic/Research	148 (25)	413 (30)	858 (40)	1705 (42)	505 (17)	735 (29)	379 (27)	909 (30)
Integrated Network	96 (16)	203 (15)	249 (12)	472 (12)	305 (10)	271 (11)	202 (14)	385 (13)
Unknown	37 (6)	91 (7)	192 (9)	315 (8)	1249 (41)	505 (20)	75 (5)	180 (6)
**Facility Location**	**0.025**		**0.003**		** < 0.001**		** < 0.001**	
Northeast	109 (18)	312 (23)	440 (21)	815 (20)	414 (14)	528 (21)	277 (20)	609 (20)
Midwest	152 (25)	365 (27)	420 (20)	969 (24)	376 (12)	556 (22)	323 (23)	755 (25)
South	202 (34)	431 (32)	753 (35)	1375 (34)	613 (20)	668 (26)	507 (36)	1102 (37)
West	101 (17)	170 (12)	341 (16)	599 (15)	393 (13)	301 (12)	241 (17)	355 (12)
Unknown	37 (6)	91 (7)	192 (9)	315 (8)	1249 (41)	505 (20)	75 (5)	180 (6)
**AJCC Stage**	** < 0.001**		** < 0.001**		** < 0.001**		** < 0.001**	
1	140 (23)	204 (15)	189 (9)	308 (8)	2381 (78)	945 (37)	261 (18)	471 (16)
2	376 (63)	861 (63)	633 (30)	1313 (32)	339 (11)	705 (28)	509 (36)	1064 (36)
3	31 (5)	160 (12)	156 (7)	522 (13)	177 (6)	593 (23)	180 (13)	605 (20)
4	50 (8)	142 (10)	1096 (51)	1871 (46)	145 (5)	313 (12)	320 (22)	757 (25)
0	4 (1)	2 (< 1)	72 (3)	59 (1)	3 (< 1)	2 (< 1)	153 (11)	104 (3)
**Grade**	**0.009**		** < 0.001**		** < 0.001**		** < 0.001**	
Well Differentiated	147 (24)	283 (21)	820 (38)	1234 (30)	2028 (67)	1231 (48)	291 (20)	483 (16)
Moderately Differentiated	87 (15)	229 (17)	553 (26)	1208 (30)	212 (7)	594 (23)	660 (46)	1508 (50)
Poorly Differentiated	56 (9)	189 (14)	439 (20)	1089 (27)	163 (5)	438 (17)	292 (21)	760 (25)
Unknown	311 (52)	668 (49)	334 (16)	542 (13)	642 (21)	295 (12)	180 (13)	250 (8)
**Lymphovascular Invasion**	0.098		** < 0.001**		** < 0.001**		** < 0.001**	
Absent	380 (63)	821 (60)	1016 (47)	1925 (47)	2160 (71)	1365 (53)	782 (55)	1505 (50)
Present	100 (17)	285 (21)	244 (11)	706 (17)	275 (9)	615 (24)	237 (17)	676 (23)
Unknown	121 (20)	263 (19)	886 (41)	1442 (35)	610 (20)	578 (23)	404 (28)	820 (27)

*P*-values reflect chi-squared analyses assessing for equal distribution of the characteristic across type of operation for each histologic subtype

*Abbreviations*: *Appy* Appendectomy, *RHC* Right hemicolectomy, *AJCC* American Joint Commission on Cancer, *quar* quartiles

**Fig. 2:**
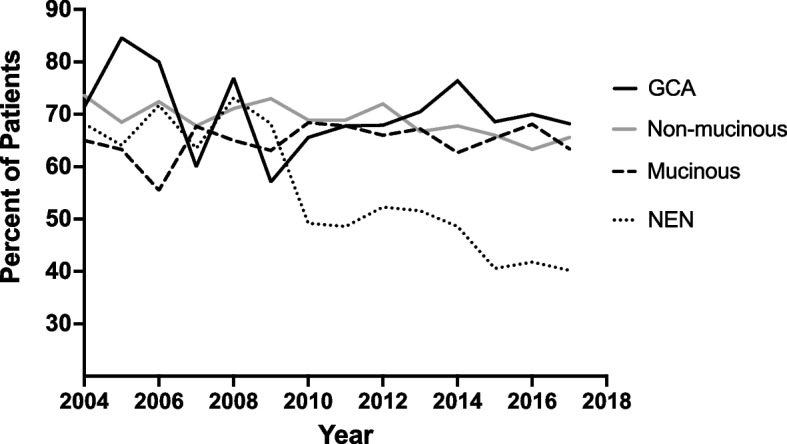
Annual proportion of patients with appendiceal cancer receiving right hemicolectomy stratified by histologic type. Abbreviations: GCA, Goblet cell adenocarcinoma; NEN, Neuroendocrine neoplasm

The trend in rate of RHC over time varied considerably by tumor type (Fig. 2). Rate of RHC was approximately constant over the study period for mucinous and non-mucinous adenocarcinoma at around 60–70%. RHC for GCA was initially labile, ranging from a peak of 85% of surgeries for GCA in 2005 to a nadir of 57% in 2009, before reaching a plateau of about 70% from 2015–2017. RHC for NEN decreased dramatically over the study period, from 68% of cases in 2004 to 40% of cases in 2017 (*p* = 0.008).

### Predictors of right hemicolectomy

Predictors of RHC varied by type of tumor (Table 2). Stage of disease and facility location were significant predictors of extent of surgery for all tumor types.

**Table 2:** Risk-adjusted odds ratios for undergoing right hemicolectomy rather than appendectomy associated with select patient, facility, and tumor characteristics

Characteristic	Goblet Cell		Mucinous		Neuroendocrine		Non-mucinous	
	**OR (95% CI)**	*p*	**OR (95% CI)**	*p*	**OR (95% CI)**	*p*	**OR (95% CI)**	*p*
**Age**	*p* = 0.784		*p* = 0.522		***p* = 0.006**		***p* = 0.031**	
< 65	Ref	-	Ref	-	Ref	-	Ref	-
≥ 65	1.0 (0.8, 1.4)	0.784	1.1 (0.9, 1.2)	0.522	**1.3 (1.1, 1.6)**	**0.006**	**0.8 (0.7, 1.0)**	**0.031**
**Charlson-Deyo Score**	0.061		0.806		**0.003**		0.082	
0	Ref	-	Ref	-	Ref	-	Ref	-
1	1.3 (1.0, 1.7)	0.055	1.0 (0.9, 1.2)	0.876	**1.3 (1.1, 1.5)**	**0.008**	1.2 (1.0, 1.4)	0.068
≥ 2	1.4 (0.9, 2.3)	0.107	1.1 (0.8, 1.4)	0.514	**1.4 (1.1, 1.9)**	**0.012**	0.9 (0.7, 1.1)	0.325
**Year of Diagnosis**	0.381		**0.033**		0.081		**0.015**	
2004–2010	Ref	-	Ref	-	Ref	-	Ref	-
2011–2017	1.1 (0.8, 1.5)	0.381	**0.9 (0.7, 1.0)**	**0.033**	0.8 (0.7, 1.0)	0.081	**0.8 (0.7, 1.0)**	**0.015**
**Facility Type**	0.282		**0.054**		** < 0.001**		0.186	
Community	Ref	-	Ref	-	Ref	-	Ref	-
Comprehensive Community	1.2 (0.9, 1.6)	0.297	0.8 (0.7, 1.1)	0.128	1.0 (0.7, 1.2)	0.699	0.9 (0.7, 1.1)	0.259
Academic/Research	1.4 (1.0, 2.0)	0.052	1.0 (0.8, 1.3)	0.957	1.1 (0.8, 1.4)	0.624	1.0 (0.8, 1.3)	0.765
Integrated Network	1.1 (0.7, 1.5)	0.772	0.9 (0.6, 1.1)	0.246	0.8 (0.6, 1.0)	0.092	0.8 (0.6, 1.1)	0.190
Unknown facility type or location^a^	1.2 (0.7, 2.1)	0.450	0.9 (0.6, 1.2)	0.392	**0.5 (0.4, 0.7)**	** < 0.001**	1.0 (0.7, 1.5)	0.995
**Facility Location**	**0.037**		**0.012**		** < 0.001**		**0.001**	
Northeast	Ref	-	Ref	-	Ref	-	Ref	-
Midwest	0.8 (0.6, 1.1)	0.280	**1.3 (1.1, 1.5)**	**0.006**	1.2 (1.0, 1.5)	0.034	1.1 (0.9, 1.3)	0.515
South	0.8 (0.6, 1.0)	0.063	1.0 (0.9, 1.2)	0.817	0.9 (0.8, 1.1)	0.377	1.0 (0.8, 1.2)	0.917
West	**0.6 (0.4, 0.9)**	**0.005**	1.0 (0.9, 1.2)	0.985	**0.7 (0.6, 0.9)**	**0.004**	**0.7 (0.6, 0.9)**	**0.003**
**AJCC Stage**	** < 0.001**		** < 0.001**		** < 0.001**		** < 0.001**	
1	Ref	-	Ref	-	Ref	-	Ref	-
2	**1.5 (1.2, 2.0)**	** < 0.001**	1.2 (0.9, 1.4)	0.163	**4.2 (3.6, 5.0)**	** < 0.001**	1.1 (0.9, 1.3)	0.337
3	**3.4 (2.2, 5.4)**	** < 0.001**	**1.6 (1.2, 2.1)**	** < 0.001**	**6.8 (5.6, 8.4)**	** < 0.001**	**1.6 (1.3, 2.1)**	** < 0.001**
4	**1.7 (1.1, 2.6)**	**0.013**	1.0 (0.8, 1.2)	0.687	**3.1 (2.4, 4.0)**	** < 0.001**	1.1 (0.9, 1.4)	0.341
0	0.4 (0.1, 2.2)	0.279	**0.5 (0.4, 0.8)**	**0.002**	0.9 (0.1, 5.9)	0.940	**0.4 (0.3, 0.6)**	** < 0.001**
**Grade**	0.163		** < 0.001**		0.160		0.097	
Well Differentiated	Ref	-	Ref	-	Ref	-	Ref	-
Moderately Differentiated	1.3 (0.9, 1.8)	0.110	**1.3 (1.2, 1.5)**	** < 0.001**	**1.3 (1.0, 1.6)**	**0.032**	**1.2 (1.0, 1.5)**	**0.022**
Poorly Differentiated	1.4 (1.0, 2.1)	0.084	**1.4 (1.2, 1.7)**	** < 0.001**	1.2 (0.9, 1.5)	0.241	1.2 (1.0, 1.5)	0.087
Unknown	1.1 (0.8, 1.4)	0.686	1.1 (0.9, 1.3)	0.268	1.0 (0.9, 1.2)	0.676	1.0 (0.8, 1.3)	0.920
**Lymphovascular Invasion**	0.993		** < 0.001**		** < 0.001**		0.051	
Absent	Ref	-	Ref	-	Ref	-	Ref	-
Present	1.0 (0.8, 1.3)	0.910	**1.3 (1.1, 1.5)**	**0.012**	**1.5 (1.3, 1.8)**	** < 0.001**	1.2 (1.0, 1.4)	0.140
Unknown	1.0 (0.8, 1.3)	0.962	**0.8 (0.7, 1.0)**	**0.010**	1.1 (0.9, 1.2)	0.457	0.9 (0.7, 1.1)	0.153

Model was additionally adjusted for sex, patient ethnicity, patient race, insurance status, and facility urbanicity

*P*-values associated with inclusion of the variable as a whole in the model are displayed in the same row as the variable name

*Abbreviations*: *OR* Odds ratio, *CI* Confidence interval, *Ref* Reference group, *AJCC* American Joint Commission on Cancer

^a^All cases with unknown facility type also had unknown facility location and vice versa, so these odds ratios pertain to both unknown facility type and unknown facility location

For GCA, cases treated in the West were 40% less likely to undergo RHC than cases treated in the Northeast (OR: 0.6, 95% CI: 0.4–0.9, *p* = 0.005). Stage 2 (OR: 1.5, 95% CI: 1.2–2.0, *p* < 0.001), stage 3 (OR: 3.4, 95% CI: 2.2–5.4, *p* < 0.001), and stage 4 (OR: 1.7, 95% CI: 1.1–2.6, *p* = 0.013) disease were significantly more likely to be treated with RHC than stage 1 tumors.

For mucinous adenocarcinoma, cases treated in the Midwest were 30% more likely to receive RHC than those treated in the Northeast (OR: 1.3, 95% CI: 1.1–1.5, *p* = 0.006). Stage 3 cases were 60% more likely to be treated with RHC (OR: 1.6, 95% CI: 1.2–2.1, *p* < 0.001) compared to stage 1 cases. Moderately differentiated (OR: 1.3, 95% CI: 1.2–1.5, *p* < 0.001) and poorly-differentiated (OR: 1.4, 95% CI: 1.2–1.7, *p* < 0.001) tumors were more likely to undergo RHC than well-differentiated tumors. Tumors with lymphovascular invasion were also significantly more likely to undergo RHC (OR: 1.3, 95% CI: 1.1–1.5, *p* = 0.012), as were cases diagnosed in 2011 or after rather than before.

For NEN, patients with age ≥ 65 were more likely to undergo RHC (OR: 1.3, 95% CI: 1.1–1.6, *p* = 0.006), as were those with a Charlson-Deyo score of 1 (OR: 1.3, 95% CI: 1.1–1.5, *p* = 0.008) or ≥ 2 (1.4, 95% CI: 1.1–1.9, *p* = 0.012) rather than 0. Cases treated in the West were 30% less likely to undergo RHC than cases treated in the Northeast (OR: 0.7, 95% CI: 0.6–0.9, *p* = 0.004). Stage 2 (OR: 4.2, 95% CI: 3.6–5.0, *p* < 0.001), stage 3 (OR: 6.8, 95% CI: 5.6–8.4, *p* < 0.001), and stage 4 (OR: 3.1, 95% CI: 2.4–4.0, *p* < 0.001) tumors were significantly more likely to be treated with RHC than stage 1 tumors. Moderately differentiated (OR: 1.3, 95% CI: 1.0–1.7, *p* = 0.032) tumors and tumors with lymphovascular invasion (OR: 1.5, 95% CI: 1.3–1.8, *p* < 0.001) were significantly more likely to be treated with RHC.

For non-mucinous adenocarcinoma, patients diagnosed 2011–2017 were 20% less likely to undergo RHC than those diagnosed 2004–2010 (OR: 0.8, 95% CI: 0.7–1.0, *p* = 0.015). Cases treated in the West were 30% less likely to undergo RHC than cases treated in the Northeast (OR: 0.7, 95% CI: 0.6–0.9, *p* = 0.003). Stage 3 tumors (OR: 1.6, 95% CI: 1.3–2.1, *p* < 0.001) were significantly more likely to be treated with RHC than stage 1 tumors. Moderately differentiated tumors were also significantly more likely to undergo RHC (OR: 1.2, 95% CI: 1.0–1.5, *p* = 0.022).

### Patient outcomes and survival analysis by type of operation

Surgical outcomes of appendectomy and RHC stratified by tumor type and stage are presented in Table 3. Negative surgical margins were more common after RHC than after appendectomy for all stages and all tumor types, although the difference did not reach statistical significance for some smaller subgroups. The disparity in overall negative surgical margin rate was greatest for stage 2 disease, in which appendectomy yielded negative margins in 83% of cases (all cancer types) compared to 93% for RHC cases (*p* < 0.001). For stage 3 GCA, appendectomy resulted in negative margins in only 61% of cases, compared to 86% of RHC cases (*p* = 0.003).

**Table 3:** Patient outcomes stratified by histologic subtype and operation

Characteristic	Goblet Cell n (%)		Mucinous n (%)		Neuroendocrine n (%)		Non-mucinous n (%)		Overall n (%)	
	**Appy**	**RHC**	**Appy**	**RHC**	**Appy**	**RHC**	**Appy**	**RHC**	**Appy**	**RHC**
**Surgical Margins S1**	*p* = 0.056		***p* < 0.001**		***p* = 0.015**		***p* < 0.001**		***p* < 0.001**	
Negative	127 (91)	197 (97)	170 (90)	303 (98)	2278 (96)	924 (98)	242 (93)	464 (98)	2817 (95)	1888 (98)
Positive	7 (5)	5 (3)	9 (5)	4 (1)	45 (2)	10 (1)	13 (5)	3 (1)	74 (2)	22 (1)
Not Evaluable or Unk	6 (4)	2 (1)	10 (5)	1 (< 1)	58 (2)	11 (1)	6 (2)	4 (1)	80 (3)	18 (1)
**Surgical Margins S2**	** < 0.001**		** < 0.001**		** < 0.001**		** < 0.001**		** < 0.001**	
Negative	321 (85)	831 (97)	504 (80)	1164 (89)	301 (89)	682 (97)	415 (82)	978 (92)	1541 (83)	3655 (93)
Positive	39 (10)	22 (3)	78 (12)	100 (7)	30 (9)	20 (3)	74 (15)	72 (7)	221 (12)	214 (5)
Not Evaluable or Unk	16 (4)	8 (1)	51 (8)	49 (4)	8 (2)	3 (< 1)	20 (4)	14 (1)	95 (5)	74 (2)
**Surgical Margins S3**	**0.003**		0.090		0.425		0.216		**0.008**	
Negative	19 (61)	138 (86)	120 (77)	418 (80)	152 (86)	530 (89)	139 (77)	502 (83)	430 (79)	1588 (85)
Positive	11 (36)	19 (12)	28 (18)	94 (18)	20 (11)	49 (8)	35 (19)	88 (15)	94 (17)	250 (13)
Not Evaluable or Unk	1 (3)	3 (2)	8 (5)	10 (2)	5 (3)	14 (2)	6 (3)	15 (2)	20 (4)	42 (2)
**Surgical Margins S4**	0.146		** < 0.001**		**0.039**		0.087		** < 0.001**	
Negative	18 (36)	74 (52)	486 (44)	921 (49)	65 (45)	180 (58)	159 (50)	419 (55)	728 (45)	1594 (52)
Positive	23 (46)	49 (35)	405 (37)	699 (37)	68 (47)	111 (35)	124 (39)	278 (37)	620 (39)	1137 (37)
Not Evaluable or Unk	9 (18)	19 (13)	205 (19)	251 (13)	12 (8)	22 (7)	37 (12)	60 (8)	263 (16)	352 (11)
**Surgical Margins All Stages**	** < 0.001**		** < 0.001**		**0.001**		** < 0.001**		** < 0.001**	
Negative	489 (81)	1242 (91)	1351 (63)	2864 (70)	2799 (92)	2317 (91)	1098 (77)	2464 (82)	5737 (80)	8887 (81)
Positive	80 (13)	95 (7)	520 (24)	898 (22)	163 (5)	191 (7)	249 (18)	442 (15)	1012 (14)	1626 (15)
Not Evaluable or Unk	32 (5)	32 (2)	275 (13)	311 (8)	83 (3)	50 (2)	76 (5)	95 (3)	466 (6)	488 (4)
**30-day Mortality**	0.896		0.747		**0.049**		0.636		0.371	
Alive	494 (99)	1143 (99)	1856 (99)	3579 (99)	2219 (99)	2051 (99)	1189 (98)	2562 (98)	5758 (99)	9335 (98)
Deceased	4 (1)	10 (1)	28 (1)	50 (1)	19 (1)	31 (1)	29 (2)	56 (2)	80 (1)	147 (2)
Unknown^a^	103 (17)	216 (16)	262 (12)	444 (11)	807 (27)	476 (19)	205 (14)	383 (13)	1377 (19)	1519 (14)
**90-day Mortality**	0.857		0.333		**0.004**		0.210		0.520	
Alive	485 (99)	1129 (99)	1813 (97)	3508 (97)	2174 (99)	2015 (97)	1150 (95)	2501 (96)	5622 (97)	9153 (97)
Deceased	7 (1)	15 (1)	64 (3)	106 (3)	33 (1)	57 (3)	62 (5)	110 (4)	166 (3)	288 (3)
Unknown^a^	109 (18)	225 (16)	269 (13)	459 (11)	838 (28)	486 (19)	211 (15)	390 (13)	1427 (20)	1560 (14)
**Post-Operative Stay**	0.891		0.357		** < 0.001**		0.994		** < 0.001**	
Median (Interquartile Range)	3 (1, 6)	4 (3, 6)	5 (2, 8)	6 (4, 9)	1 (0, 4)	4 (3, 6)	5 (2, 8)	5 (4. 7)	3 (1, 6)	5 (3, 8)
**Readmission within 30 Days**	**0.002**		** < 0.001**		** < 0.001**		0.781		** < 0.001**	
No Readmission	554 (92)	1243 (91)	1940 (90)	3679 (90)	2897 (95)	2345 (92)	1289 (91)	2689 (90)	6680 (93)	9956 (91)
Unplanned Readmission	17 (3)	82 (6)	75 (3)	217 (5)	91 (3)	124 (5)	69 (5)	164 (5)	252 (4)	587 (5)
Planned Readmission	20 (3)	36 (3)	56 (3)	88 (2)	38 (1)	54 (2)	38 (3)	88 (3)	152 (2)	266 (2)
Unknown	10 (2)	8 (1)	75 (3)	89 (2)	19 (1)	35 (1)	27 (2)	60 (2)	131 (2)	192 (2)

*P*-values reflect chi-squared analyses and a two-tailed independent samples t-test assessing for association between outcome and type of operation for each histologic type

*Abbreviations*: *Appy* Appendectomy, *RHC* Right hemicolectomy, *S* Stage, *Unk* Unknown

^a^Cases with vital status unknown at 30 days and 90 days (including all cases diagnosed in 2017) were excluded from the analysis

RHC was also associated with longer inpatient stays compared to appendectomy for the group as a whole (median 5 days vs. 3 days, *p* < 0.001). A higher proportion of RHC cases resulted in unplanned readmission for all tumor types except non-mucinous adenocarcinoma and for the study group as a whole. For patients with NEN, RHC was associated with higher 30-day mortality (1.2% vs. 0.6%, *p* = 0.049) and 90-day mortality (3% vs. 1%, *p* = 0.004).

**Fig. 3:**
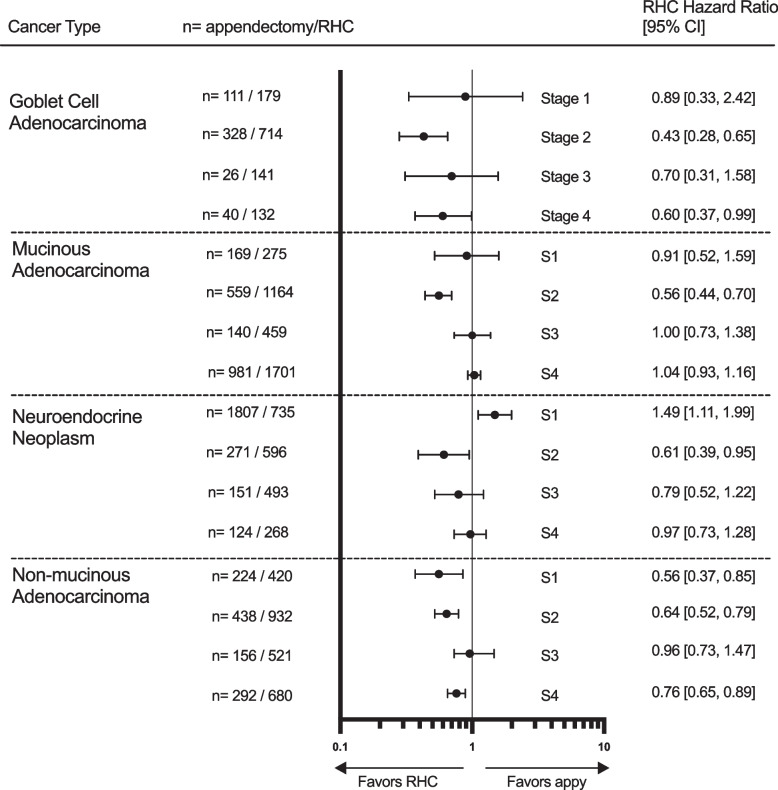
Risk-adjusted hazard ratios associated with right hemicolectomy rather than appendectomy for each stage of each histologic type of appendiceal cancer. Abbreviations: RHC, Right hemicolectomy; CI, Confidence interval; S, Stage; appy, appendectomy

On risk-adjusted survival analysis, patients who underwent RHC experienced statistically significant longer overall survival than patients who underwent appendectomy for stage 2 disease no matter the histologic type, with the strongest benefit observed in GCA (HR: 0.43, 95% CI: 0.28–0.65, *p* < 0.001) and the least benefit observed in NEN (HR: 0.61, 0.39–0.95, *p* = 0.030). A mortality benefit with RHC was also seen in stage 1 and stage 4 non-mucinous adenocarcinoma (Fig. 3). Of note, patients with stage 1 NEN who underwent RHC had a significantly *higher* mortality than those that underwent appendectomy only (HR: 1.49, 1.11–1.99, *p* = 0.007).

## Discussion

This is the first study in a national cohort to assess trends in surgical treatment of the most common histologic types of appendiceal tumors over time. We found that the rate of RHC for GCA, non-mucinous adenocarcinoma, and mucinous adenocarcinoma has been relatively constant, representing around two-thirds to three-fourths of all surgical treatments for these tumors between 2010 and 2017. The rate of RHC for appendiceal NEN, in contrast, is on a steep downward trend, with RHC representing only two-fifths of surgical treatments of NEN in 2017, compared to over 70% in earlier years. We also uncovered consistencies in factors associated with RHC across different tumor types with respect to stage, tumor grade, and lymphovascular invasion, as well as some geographic disparities in management. Finally, we describe worse short-term surgical outcomes in terms of length of stay and unplanned readmissions after RHC than after appendectomy for these diseases, but observe that stage 2 tumors treated with RHC experience longer risk-adjusted survival than those treated with appendectomy no matter the tumor type.

One striking finding with respect to the overall trends for RHC over time is that the rate of RHC for the three non-NEN tumor types appears to have reached a plateau at around 60–75% of cases, despite prominent national and international guidelines encouraging RHC in nearly all instances for all three of these cancers [9, 10, 23]. This finding suggests that clinicians that treat these diseases give credence to the studies and experts that have challenged these guidelines and recommended a less aggressive approach in some instances [11–14, 24–27] and highlights the current complexity of the literature. The decreasing rate of RHC for NENs over time reflects a growing body of evidence and the recommendation of all major guidelines that appendectomy is sufficient for low-risk tumors of this type [9, 10, 23, 28] and demonstrates that these recommendations are currently still in the process of being adopted.

With respect to predictors of surgical treatment, we found that stage of disease held the strongest association with RHC for all cancer types. This association was largest in magnitude for NENs, suggesting that the size-driven NANETS, ENETS, and NCCN guidelines for the management of this disease have had some influence on clinical decision-making [10, 23]. This interpretation is supported by a previous study which demonstrated that about two-thirds of patients with appendiceal NEN receive care adherent with NCCN guidelines [19]. While that study failed to find an association between guideline-adherent care and survival, our study suggests that patients with stage II (tumor size 2–4 cm) NEN do experience longer survival after RHC than after appendectomy (HR: 0.61). Our results also suggest that stage 3 disease is associated with RHC for all tumor types; however, it is difficult to interpret this finding in the context of this retrospective analysis, as the diagnosis of stage 3 disease – which is characterized by lymph node involvement – is much more likely to be made after RHC, which tends to yield numerous lymph nodes, than after appendectomy, during which lymph nodes are rarely harvested [21]. Two other tumor characteristics that were associated with RHC in our study were higher grade and lymphovascular invasion, which are known prognostic markers for these cancers [10, 19, 20].

Our study also highlights variability in practice patterns across the country. Even after adjustment for 13 patient, tumor, and facility variables, patients with GCA, NEN and non-mucinous adenocarcinoma treated in the West were 30–40% less likely to undergo RHC than those treated in the Northeast. The driving force behind this disparity is unclear and warrants further investigation. One recent study found that patients in the West and the South have an average further distance to the nearest providers of colorectal and cervical cancer care [29], while another found that New England had the highest concentration of oncologists of any region in the United States [30], suggesting that access to specialists may contribute to these disparities. It is also conceivable that institutional-level oncology protocols contribute to this trend. Variability in coverage provided by regional insurance plans and other unmeasured differences in the regional patient populations are other possible drivers of these geographic disparities.

Another contribution of this analysis to the literature is the examination of short-term postsurgical outcomes. We found RHC to be associated with a 5–10% higher rate of negative surgical margins than appendectomy for all cancer types and stages, similar to previously published findings [25, 31]. Appendectomy, on the other hand, was associated with a median postoperative hospital stay two days shorter than that after RHC, which has implications extending to cost of care, risk of hospital-acquired conditions, and lost wages. In addition, appendectomy was associated with fewer unplanned 30-day readmissions than RHC. This may be due to a shorter hospital stay, discharge to home rather than a nursing facility, or decreased likelihood of ostomy or blood transfusion, all of which have been previously associated with readmission after colectomy for cancer [32]. Importantly, readmission after colectomy has also previously been found to be an independent predictor of one-year mortality [32]. These findings underscore the value to patients and the healthcare system of avoiding nonbeneficial RHC if a negative margin can reasonably be obtained with appendectomy.

While multiple previous studies have compared survival after RHC to survival after appendectomy for these types of appendix cancer [11, 14, 19, 25–27, 31, 33, 34], our analysis uniquely allows for direct comparisons in survival benefit of RHC by stage across different tumor types. One of the most striking findings of our study is that RHC seems to offer survival benefit for stage 1, 2, and 4 non-mucinous adenocarcinoma, reinforcing current guidelines to perform a RHC in all cases for this diagnosis [9]. We also found that RHC is associated with improved survival for stage 2 disease no matter the histology, offering further evidence that size of the primary tumor may be a reliable marker for guiding surgical treatment – one that can be assessed noninvasively via cross sectional imaging rather than requiring a tissue diagnosis. Last, we found that RHC is associated with *worse* survival for stage 1 appendiceal NEN. This may be driven by the elevated 30- and 90-day mortality after RHC compared to appendectomy for this disease, and suggests that appendectomy should be the initial approach for small tumors if NEN is suspected. It is possible that this elevated mortality is in part due to an overrepresentation of more the lethal high-grade neuroendocrine carcinomas in the stage 1 RHC group compared to the stage 1 appendectomy group, but we attempted to account for this by adjusting for grade in our analysis.

There are several limitations to our study. First, this is a retrospective database and all limitations inherent to retrospective studies apply; these have been well-described elsewhere [35]. Only patients treated at Commission on Cancer-accredited facilities are captured by the NCDB, so our results are not generalizable to many community centers which may treat these diseases. We also did not control for oncologic therapies other than surgical resection of the primary tumor such as cytoreductive surgery or systemic or intraperitoneal chemotherapy, which could impact survival outcomes for some of these types of cancer. Last, the decision to assess diverse types of tumors offers benefits with respect to comparisons between tumor types, but limits our ability to adjust for prognostic factors that are unique to one histologic type or another.

## Conclusion

Around 60–70% of patients with appendiceal GCA, mucinous adenocarcinoma, or non-mucinous adenocarcinoma undergo RHC, and this rate has been relatively constant in recent years. Around 40% of patients with appendiceal NEN undergo RHC, and this rate has been decreasing. RHC is overall associated with increased rates of readmission and longer postoperative length of stay than appendectomy, but RHC is associated with a risk-adjusted survival benefit for stage 2 disease of any histology and for stage 1 and 4 non-mucinous adenocarcinoma. For stage I NEN, RHC is associated with higher risk-adjusted mortality. Guidelines for the surgical management of appendiceal tumors should be simplified or consolidated and should take into consideration the short-term and long-term outcomes associated with appendectomy and RHC.

## Data Availability

The data that support the findings of this study are available from National Cancer Database (NCDB) but restrictions apply to the availability of these data, which were used under license for the current study, and so are not publicly available. Data are however available from the corresponding author, Dr. Sajid Khan, upon reasonable request and with permission of the American College of Surgeons National Cancer Database.
